# Therapeutics for sickle cell disease intravascular hemolysis

**DOI:** 10.3389/fphys.2024.1474569

**Published:** 2024-09-13

**Authors:** Jianyao Xue, Xiang-An Li

**Affiliations:** ^1^ Department of Pharmacology and Nutritional Sciences, University of Kentucky College of Medicine, Lexington, KY, United States; ^2^ Lexington VA Healthcare System, Lexington, KY, United States; ^3^ Department of Physiology, University of Kentucky College of Medicine, Lexington, KY, United States; ^4^ Saha Cardiovascular Research Center, University of Kentucky College of Medicine, Lexington, KY, United States

**Keywords:** sickle cell disease, hemolysis, heme toxicity, scavenging receptors, transfusion

## Abstract

Sickle cell disease (SCD) is a genetic disorder predominantly affecting individuals of African descent, with a significant global health burden. SCD is characterized by intravascular hemolysis, driven by the polymerization of mutated hemoglobin within red blood cells (RBCs), leading to vascular inflammation, organ damage, and heme toxicity. Clinical manifestations include acute pain crises, hemolytic anemia, and multi-organ dysfunction, imposing substantial morbidity and mortality challenges. Current therapeutic strategies mitigate these complications by increasing the concentration of RBCs with normal hemoglobin via transfusion, inducing fetal hemoglobin, restoring nitric oxide signaling, inhibiting platelet-endothelium interaction, and stabilizing hemoglobin in its oxygenated state. While hydroxyurea and gene therapies show promise, each faces distinct challenges. Hydroxyurea’s efficacy varies among patients, and gene therapies, though effective, are limited by issues of accessibility and affordability. An emerging frontier in SCD management involves harnessing endogenous clearance mechanisms for hemolysis products. A recent work by Heggland et al. showed that CD-36-like proteins mediate heme absorption in hematophagous ectoparasite, a type of parasite that feeds on the blood of its host. This discovery underscores the need for further investigation into scavenger receptors (e.g., CD36, SR-BI, SR-BII) for their possible role in heme uptake and detoxification in mammalian species. In this review, we discussed current SCD therapeutics and the specific stages of pathophysiology they target. We identified the limitations of existing treatments and explored potential future developments for novel SCD therapies. Novel therapeutic targets, including heme scavenging pathways, hold the potential for improving outcomes and reducing the global burden of SCD.

## Introduction

Sickle cell disease (SCD) is a genetic disorder that disproportionally affects people of African descent (Luzzatto, 2012). In central Africa, sickle hemoglobin allele frequency can reach up to 18% (Grosse et al., 2011). In central Africa, a mortality rate exceeding 50% is observed among children with sickle cell disease, with fatalities typically occurring before they reach the age of five (Grosse et al., 2011). In recent years, the migration of people with the sickle-cell allele from Africa and India to Western Europe, the eastern coast of South and North America has increased (Inusa et al., 2019). Subsequently, incidence of the disease where the disorder was not previously documented is on the rise (Piel et al., 2013). In the United States, it is estimated that 100,000 people are affected by SCD and one in 500 African American babies are born with this condition (Pleasants, 2014). Globally, projections estimate a surge of over 25% by 2050, leading to an annual count of 400,000 infants born with the disorder (Pleasants, 2014).

Intravascular hemolysis, a critical contributor to vaso-occlusive crises---the principal pathological manifestation of SCD---refers to the destruction of red blood cells (RBCs) within the blood vessels (Dutra and Bozza, 2014; Kato et al., 2018; Kato et al., 2017). The destruction of RBCs releases their intracellular contents and initiates a cascade of events leading to vascular inflammation, organ damage, and heme toxicity (Van Avondt et al., 2019). Heme, a component of hemoglobin (Hb) in the RBCs, is highly toxic when released into the bloodstream in large amounts (Belcher et al., 2014). High levels of heme can induce oxidative stress, activate inflammatory pathways, and cause direct damage to cells, tissues, and organs (Saraf, 2020). This can result in a range of clinical complications, such as hemolytic anemia, thrombotic events, acute pain crises, acute kidney injury, and multi-organ dysfunction (Smith and McCulloh, 2015). Among these complications, acute pain due to vaso-occlusion is the most common reason for emergency department visits of SCD patients (Yusuf et al., 2010; Tanabe et al., 2019).

### SCD pathophysiology

In sickle cell disease, beta-globin, a component making up 50% of the Hb, has a genetic mutation. This mutation changes the sixth amino acid from glutamic acid (hydrophilic) to valine (hydrophobic). Consequentially, delicate interactions among amino acids are altered. This causes mutated Hb to polymerize within RBCs after oxygen unloading (Kato et al., 2018). Hb is the most abundant protein in RBC, taking up 97% of the cell dry weight (Kuhn et al., 2017). The polymerization of Hb changes the shape of RBC from a round disc to a sickle (Eaton and Bunn, 2017). The sickling of red blood cells diminishes their ability to deform and pass through capillary beds, leading to ischemia and reperfusion injuries (Rees et al., 2010). This unfavorable shape change also increases RBCs susceptibility to hemolysis and reduces RBCs lifespan. Due to frequent hemolysis, sickle RBCs only have a lifespan of 10–20 days, in stark contrast to the 120 days of normal RBCs in a healthy individual (Steinberg, 2008). As the RBCs break open, the intracellular contents are released into the bloodstream, triggering pathological events.

The RBC intracellular contents can be categorized into three groups, all of which contribute to vaso-occlusive pain attacks. The first group reduces the bioavailability of nitric oxide (NO), an important molecule for the relaxation of the blood vessels. This group includes liberated Hb and an enzyme called arginase 1^8^. Arginase one could deplete circulating arginine, a substrate that is required by endothelial nitric oxide synthase (eNOS) for NO production (Kato et al., 2018). The depletion of arginine, thus, leads to decreased NO bioavailability. On the other hand, Hb can directly scavenge NO through a dioxygenation reaction, thereby reducing NO bioavailability (Kato et al., 2017). Physiologically, nitric oxide (NO) generated by endothelial cells diffuses into nearby smooth muscle cells, where it activates soluble guanylate cyclase (sGC). This activation catalyzes the production of cyclic guanosine monophosphate (cGMP). cGMP then signals for vasorelaxation (Stasch et al., 2011). Because of the important role of NO in blood vessel relaxation, decreased NO bioavailability contributes to vasoconstriction and endothelial dysfunction seen in SCD (Murad, 2006).

The second group of RBC intracellular contents activates platelets. It includes ATP/ADP that are released from lysed RBCs. These molecules activate P2Y12 receptors on the platelets and stimulate the release of platelet granule contents (Dorsam and Kunapuli, 2004). The release of granule contents significantly enhances the risk of blood clots. Research indicates that adults with SCD have a significantly higher risk of venous thromboembolism (VTE). Specifically, they have a 4- to 100-fold increased risk for VTE and a 2- to 4-fold increased risk of VTE-related death compared to the general population (Davila et al., 2023). A recent work by Nolfi-Donegan et al. demonstrated that high-mobility group box 1(HMGB1), a DNA-binding protein that is released by damaged tissue and platelets, strongly enhances the ADP-P2Y12 platelet activation pathway in sickle cell disease (Nolfi-Donegan et al., 2024). As platelets become activated and adhesive, the risk of vaso-occlusion, tissue ischemia, and pain crisis increase, especially when coupled with vasoconstriction due to decreased NO availability (Downing et al., 1997).

The third group triggers a potent inflammatory response. It includes liberated Hb and heme. Normal Hb exists as a tetramer. Liberated Hb, however, will quickly dissociate into dimers (Vallelian et al., 2022). The Hb dimers could infiltrate renal tubules and cause renal damage (Schaer et al., 2013). Liberated Hb will initiate Fenton reactions to generate free radicals (Kato et al., 2017). Upon oxidation reactions with radicals or NO, Hb releases its’ prosthetic group, heme (Balla et al., 1991). Heme is a potent inflammatory molecule that activates the innate immune system and endothelial cells, leading to an inflammatory and pro-adhesive state (Wagener et al., 2001). In a humanized mice model of SCD, Belcher et al. demonstrated that the liberation of heme from Hb is a necessary step towards vaso-occlusion (Belcher et al., 2014). In SCD, approximately one-third of RBCs are lysed within the vasculature (Crosby, 1955). This process liberates a large amount of Hb and heme, causing pain crises in SCD patients (Kato et al., 2018; Saraf, 2020; Smith and McCulloh, 2015).

### Therapeutics for SCD

**FIGURE 1:**
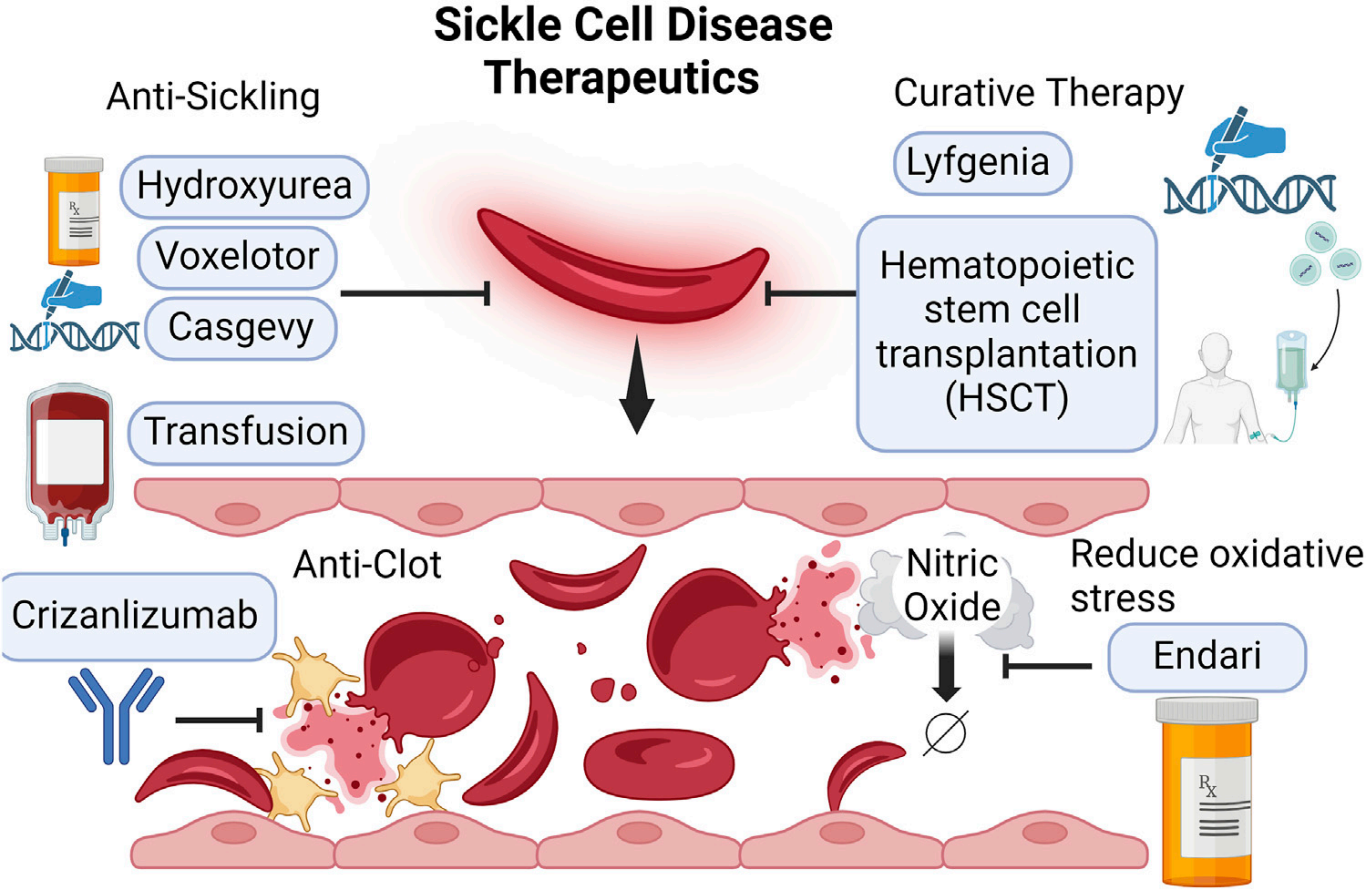
Currently available SCD therapeutics. SCD severity is driven by the sickling of the RBCs and subsequent intravascular hemolysis. Hydroxyurea and gene therapy Casgevy induce fetal Hb production, mitigating sickling severity. Voxelotor prevents sickling by stabilizing Hb in the oxygenated state. Crizanlizumab, a P-selectin monoclonal antibody, blocks the interaction between P-selectin and P-selectin receptors on the activated platelets (yellow) and endothelium. This reduces the risk for clots and vaso-occlusion. Transfusion may improve oxygen delivery and dilute out the number of sickle cells. L-glutamine may increase NO bioavailability and reduce oxidative stress. The only two curative therapies for SCD are hematopoietic stem cell transplantation (HSCT) and gene therapy Lyfgenia.

Therapeutic approaches have been developed to target each aspect of the sickle cell disease pathophysiology. The first arena has focused on the induction of fetal hemoglobin (HbF) to alleviate disease severity. Currently, the first line of treatment is hydroxyurea (Figure 1). Approved by FDA in 1997, hydroxyurea, a ribonucleotide reductase inhibitor, can induce fetal Hb production (Ault, 1998; Musialek and Rybaczek, 2021). During gestation, fetus expresses gamma globin, which makes up fetal Hb. After birth, gamma-globin gene expression is suppressed and beta-globin expression is switched on to make adult Hb (Schechter, 2008). In 1948, Dr. Janet Watson, a pediatrician from Brooklyn, published the *significance of the paucity of sickle cells in newborn Negro infants*. She noticed that infants with SCD had less sickle red blood cells than when they became adults. She pointed to high HbF in infants might be the protective factor (Akinsheye et al., 2011). Later studies revealed that increased HbF levels could slow down the polymerization of mutated adult Hb, thereby giving the RBCs more time to leave the tighter microvasculature before sickling (Schechter, 2008; Henry et al., 2020). The induction of HbF is the principal protective mechanism by hydroxyurea. Additionally, other protective mechanisms of hydroxyurea have been proposed, including reducing adhesive reticulocytes, increasing NO production, and raising RBC volume (MCV) (Musialek and Rybaczek, 2021; King, 2003; Borba et al., 2003). Although hydroxyurea is currently the most affordable and available treatment, it does not cover all SCD patients. About 25% of SCD patients do not respond to or tolerate hydroxyurea (Sever et al., 2014). Despite being the first line of treatment, it cannot completely prevent pain attacks (Sever et al., 2014).

In the same arena of fetal Hb induction, another successful approach is the recent FDA approval of gene therapy crisper-cas9 gene editing (Casgevy) (Figure 1). This therapy increases fetal Hb production by targeting *BCL11A* erythroid-specific enhancers to reactivate the expression of gamma-globin that makes up fetal Hb (Canver, 2015). This method has demonstrated encouraging outcomes by increasing HbF and improving the clinical prognosis for patients with sickle cell disease (Frangoul et al., 2021). Compared to hydroxyurea which requires daily intake, Casgevy offers a long-term resolution. However, the current treatment cost is very high, priced at approximately $1.8 million (Jiao et al., 2021).

The second arena focused on reducing oxidative stress. L-glutamine has been shown to decrease painful crises and reduce hospital visits in a randomized, placebo-controlled trial (Niihara et al., 2018). This led to the FDA approval of L-glutamine as a treatment in 2017, making it the second drug approved to treat sickle cell disease after hydroxyurea (Minniti, 2018) (Figure 1). The mechanisms of action are believed to involve glutamine’s role as a substrate for NO synthesis, glutathione synthesis, NAD(H), and NADP(H) synthesis (Sadaf and Quinn, 2020). Although L-glutamine can modestly reduce the frequency of pain crises in SCD patients, the price is much higher than hydroxyurea. The yearly cost of L-glutamine (Endari) treatment is approximately 24 times the cost of hydroxyurea therapy (L-Glutamine for Sickle Cell Disease, 2018). Due to the high cost, the overall efficacy and cost-effectiveness of L-glutamine therapy remain a topic of debate (Quinn, 2018). L-glutamine is currently used as a second-line treatment or as an adjunctive therapy (Casgevy and Lyfgenia, 2024).

The third arena of disease management is to lower clot risk by inhibiting platelet and endothelium interaction. SCD patients have increased thrombotic tendencies due to platelet activation (Nolfi-Donegan et al., 2024). P-selectin is a cell adhesion protein and it is expressed on activated endothelium and platelets (Auvinen et al., 2014). It mediates the rolling of platelets and leukocytes on the activated endothelial cells and the formation of a stable blood clot (Merten and Thiagarajan, 2000). P-selectin inhibitors prevent adhesion of activated red cells and leukocytes (Gutsaeva et al., 2011). In 2019, Crizanlizumab, a P-selectin inhibitor, was approved by the FDA for the prevention of vaso-occlusive crises in patients with sickle cell disease (Figure 1). Crizanlizumab is a P-selectin monoclonal antibody. Early works have shown that P-selectin antibodies block the interaction between P-selectin and its receptor CD162(PSGL-1), thereby reducing the formation of thrombus (Downing et al., 1997). Clinical trials testing Crizanlizumab efficacy have yielded promising results in which a high dose (5 mg/kg) is shown to significantly decrease the pain crisis rates (Ataga et al., 2017). Despite the promising results, a critical barrier to this treatment is the cost. The yearly cost ranges between $80,000 to $110,000 (Jiao et al., 2021; Kutlar et al., 2023; Stevens et al., 2021).

The fourth arena of disease management is to decrease RBCs sickling by stabilizing Hb in an oxygenated state. Between the early 60s and mid-70s, Dr. Beutler proposed that having sickle Hb (HbS) remain in the stabilized oxygenated state or as a carbon monoxide complex may reduce sickling. This is because sickling is the result of HbS polymerization. Polymerization happens when HbS loads off oxygen. Thus, having a certain percentage of HbS stabilized in the oxygenated state may prevent sickling thereof (Beutler, 1961; Beutler, 1975). This strategy comes with a critical trade-off. The stabilization may compromise oxygen delivery to the tissue. However, He argued that because the chief clinical symptom of SCD is vaso-occlusive pain due to sickling, the decreased oxygen release may be tolerated. Fast forward to 2019, FDA approved Voxelotor (Figure 1), a drug that binds covalently to the a-globin and stabilizes Hb in the oxygenated form (Henry et al., 2021). Clinical trials have proven Dr. Beutler to be right, as this strategy has successfully reduced hemolysis markers and increased hemoglobin levels in SCD patients (Vichinsky et al., 2019). However, to many a surprise, the treatment did not result in meaningful decreases in the frequency of vaso-occlusive pain experienced by SCD patients. Meanwhile, the cost of this treatment is much higher than hydroxyurea. Compared to the cost of hydroxyurea at $100 per month, the current cost of voxelotor treatment is approximately $2000 per month (Han et al., 2020). This has made voxelotor far less cost-effective than hydroxyurea based on the price per pain crisis averted (Jiao et al., 2021; Henry et al., 2021).

Lastly, blood transfusion represents a critical intervention in preventing neurological complications in children. Transfusion may improve oxygen delivery and dilute out the number of sickle cells, and thus is beneficial in certain conditions (Rees et al., 2018). The Stroke Prevention Trial in Sickle Cell Anemia (STOP), the follow-up STOP2, and the Silent Cerebral Infarct Transfusion (SIT) trial demonstrated that chronic transfusion reduces stroke risks (Lee et al., 2006; Adams et al., 2005; DeBaun et al., 2014). Due to the challenge of the commitment to lifelong blood transfusion, the TWiTCH trial evaluated switching from transfusion to hydroxyurea for stroke prevention (Ware et al., 2016). This trial supported that at maximum tolerated dose, hydroxyurea is noninferior to blood transfusion. While these trials showed the benefits of transfusions in stroke prevention, their role in treating SCD acute chest syndrome is less clear (Chou et al., 2020). Transfusion is generally not recommended for uncomplicated VOCs (Han et al., 2021; Physicians, 2023; Panel, 2014).

Currently, the only two curative treatments of SCD are hematopoietic stem cell transplantation (HSCT) and Lyfgenia, another recently FDA-approved gene therapy that modifies patients’ stem cells to produce normal hemoglobin (Two Gene Therapies for Sickle Cell Disease, 2024) (Figure 1). Hematopoietic stem cell transplantation requires a matched related donor and is only available to approximately 15% of SCD patients (Dallas et al., 2013). Finding human leukocyte antigen (HLA) matched, and disease-free sibling for a SCD patient is difficult. There was an effort to explore using haploidentical donors. However, studies showed that a high graft failure rate of 43% is observed with HSCT using haploidentical donors (Bolanos-Meade et al., 2012). When using matched but unrelated donor marrow, a high incidence of graft versus host disease (GVHD) and associated mortality is observed (Shenoy et al., 2016). Therefore, GVHD and limited available donors hinder the success of hematopoietic stem cell transplantation as a curative treatment for SCD (Park and Bao, 2021). Gene therapy, using patients’ stem cells, can bypass the aforementioned limitations. Approved in 2023, Lyfgenia uses a lentiviral vector to add a working copy of beta-globin to patients’ stem cells and then bring them back to patients through IV infusion (Casgevy and Lyfgenia, 2024). A single-arm trial of 36 patients showed that 91% of patients have a complete absence of vaso-occlusive pain crisis 6–18 months post-engraftment (Casgevy and Lyfgenia, 2024). The current treatment cost for Lyfgenia is $3.1 million, which is the highest among all available treatments.

### Other potential therapeutics

The final frontier in disease management is harnessing the power of our body’s scavenging-clearance systems. These systems effectively remove hemolytic products (liberated Hb and heme), representing a largely untapped resource with great potential. Two mechanisms have been identified to remove Hb and heme. Both mechanisms demand the presence of scavenging proteins for effective cellular uptake: the haptoglobin-CD163 pathway and the hemopexin-CD91 pathway (Kristiansen et al., 2001; Hvidberg et al., 2005). With a strong binding affinity (∼1p.m.), haptoglobin (Hp) effectively scavenges the dimeric Hb in the bloodstream (Tolosano et al., 2002). The formation of the Hp-Hb complex prevents Hb dimers from entering the glomerulus, and this complex is then taken up by CD163+ macrophages (Haymann et al., 2010; Schaer et al., 2014). The second mechanism requires scavenging protein hemopexin (Hx). Hx has a high affinity of binding to heme (<1p.m.) (Tolosano et al., 2002). Once the Hx-heme complex forms, it is cleared by tissues and cells expressing CD91, such as the liver and macrophages (Kristiansen et al., 2001; Garland et al., 2016). Both Hp and Hx scavenge at a 1:1 stoichiometry (Tolosano et al., 2002). Following receptor-mediated endocytosis, both receptors are recycled back to the cell surface. Scavenging proteins are processed differently. In pathological conditions, such as sepsis or hemolytic crisis, Hx is partially recycled based on non-human primate studies, while Hp is eventually degraded in lysosomes (Smith and McCulloh, 2015). Limited by the available pool of these two scavenging proteins, patients experiencing severe hemolytic crises often exhibit undetectable levels of Hp and Hx in the blood, while free Hb and heme remain elevated (Saraf, 2020; Muller-Eberhard et al., 1968; Naumann et al., 1971).

Early works from mice studies have shown that both Hp and Hx play a strong protective role in hemolysis. In the late 90s, Lim et al. showed that Hp KO mice experienced greater oxidative damage and renal tubular injury compared to wild-type mice when challenged with hemolysis-inducing agents (Lim et al., 1998). A year later, Tolosano et al. showed that Hx KO mice showed more severe renal damage and iron deposition at the kidney compared to the wild-type mice (Tolosano et al., 1999). These findings highlight the important role of both haptoglobin and hemopexin in protecting against the damaging outcomes of hemolysis. In Japan, plasma-purified Hp was approved in 1985 as a treatment for hemolysis. It has been used to protect kidneys from hemolysis exclusively in acute settings such as open-heart surgery with cardiopulmonary bypass, massive blood transfusion, and burn injuries (Schaer et al., 2013). However, in chronic settings such as SCD pain management, Hp replenishing therapeutic has not been tested. Determining whether Hp could be a promising therapeutic candidate in chronic settings requires careful consideration, as Hp is rapidly consumed and eventually degraded in lysosomes (Vallelian et al., 2022). Sustaining meaningful clinical outcomes may therefore require frequent treatments. Alternatively, Hx, which has recycling properties, might be a better candidate. Currently, Hx is being investigated as a potential therapeutic in SCD in phase 1 multicenter trials with 28 patients. This study was recently completed for safety, tolerability, and pharmacokinetics of a single dose of Hx. Early mouse studies showed that Hx inhibited neutrophil migration and led to an increased septic death (Spiller et al., 2011). Thus, albeit as an important protein in scavenging heme, the safety of Hx as therapeutics warrants further investigation in clinical trials.

In addition to the scarcity of human studies testing Hx replenishment as a therapeutic in SCD, another foreseeable limitation of this strategy is the cost. Purified protein therapeutics represent some of the most expensive drugs on the market. This is due to more steps being required in manufacturing purified proteins compared to that of small molecule drugs (Lagasse et al., 2017). These factors might limit the protein treatments’ availability to SCD patients, especially for those in the under-developed area of the world.

## Future directions

Due to the imperfect therapeutic efficacy and the extremely high costs associated with current treatments, there is a pressing need for novel therapeutics. These new treatments should be both affordable and effective in completely preventing vaso-occlusive pain. However, our limited understanding of hemolysis detoxification process has hindered the development of such treatments. Therefore, it is imperative to continually invest in basic research that expands our knowledge of hemolysis and the detoxification process. Our understanding of the detoxification of hemolysis products at the receptor level remains incomplete. Studies have suggested that other clearance systems may exist, systems that do not require scavenging proteins. In 2006, Schaer et al. demonstrated that, under physiological conditions, macrophages could, uptake native Hb without the binding protein Hp through CD163 (Schaer et al., 2006). They demonstrated this uptake pathway with native and crosslinked stabilized tetrameric Hb. Whether this pathway plays an important role under pathological conditions is unknown, as CD163 has not been shown to uptake the dimer form of Hb, which is more relevant in SCD (Andersen et al., 2012). Nevertheless, this finding opens up the possibility of regulating clearance of hemolysis products at the receptor level. The CD163 receptor could be upregulated by IL-10 and glucocorticoid (GC) (Sulahian et al., 2000; Schaer et al., 2002). Schaer et al. showed that GC-treated macrophages had a higher capacity to bind and internalize Hb-Hp complex due to CD163 upregulation. GC therapy, specifically dexamethasone, has been shown to reduce vaso-occlusion in an SCD mouse model triggered by hypoxia (Belcher et al., 2005). However, clinical data have been controversial; while GC therapy could reduce the duration of opiate usage and hospitalization, patients consistently showed rebound pain readmission (Owusu-Ansah et al., 2016). Further research is needed to explore, during steady state, whether CD163 upregulation with GC and thus enhanced native Hb clearance will prevent disease progression towards a pain crisis in SCD patients.

Another early study also suggests the existence of a novel clearance pathway. An insightful study by Tolosano et al. showed that Hp Hx double knockout (DKO) mice showed a paradoxically higher survival rate than their single knockout littermates for Hx or Hp during a lethal hemolytic event (Tolosano et al., 2002). In the liver, heme oxygenase 1 (HO-1), a marker of Hb and heme uptake, was highly induced in the Kupffer cells of Hp Hx DKO mice. Since CD163 does not uptake dimeric Hb without Hp during acute hemolysis, the survival advantage and enhanced HO-1 expression may suggest novel cellular uptake routes for either Hb dimer, heme, or both. This novel pathway functions in the absence of scavenging proteins Hp and Hx, a scenario that is highly clinically relevant.

In a recent study by Heggland et al., a scavenger receptor CD-36-like protein was shown to mediate heme absorption into the intestine of hematophagous ectoparasite (Heggland et al., 2019). This suggests binding, and uptake interactions of heme with CD36 receptors, a well-conserved protein across mammalian species, may exist. In mouse liver, hepatocytes moderately express CD36 while the Kupffer cells are shown to strongly express CD36 (Yang et al., 2022). Taken together, the involvement of scavenger receptors (such as CD36, SR-B1, SR-BII) of heme uptake during hemolytic crisis warrants further investigation. The identification of a novel heme clearance pathway may open up a new frontier of treating vaso-occlusive pain in SCD.

## Conclusion

An emerging and promising approach in SCD management involves harnessing endogenous clearance mechanisms for hemolysis products. If scavenger receptors could indeed mediate the clearance of hemolysis products such as Hb and heme, then several existing drugs can be tested for their potential to prevent VOC. Existing drugs such as GC therapy, which upregulates CD163, anti-diabetic drug, pioglitazone, which upregulates CD36, and a kinase inhibitor, imatinib, which upregulates SRBI, may be evaluated for potential in regulating the clearance of hemolysis products at the receptor level (Schaer et al., 2002; Kanoke et al., 2020; Sucharski et al., 2022). By leveraging these existing therapies, we can explore new, affordable approaches to managing hemolysis, potentially improving patient outcomes and reducing healthcare costs.
